# PI3K Pathway Inhibition with NVP-BEZ235 Hinders Glycolytic Metabolism in Glioblastoma Multiforme Cells

**DOI:** 10.3390/cells10113065

**Published:** 2021-11-07

**Authors:** Shreya Udawant, Carl Litif, Alma Lopez, Bonnie Gunn, Erin Schuenzel, Megan Keniry

**Affiliations:** Department of Biology, The University of Texas Rio Grande Valley, 1201 W., Edinburg, TX 78539, USA; shreya.udawant01@utrgv.edu (S.U.); clitif@uwyo.edu (C.L.); alma.lopez03@utrgv.edu (A.L.); bonnie.gunn@utrgv.edu (B.G.); erin.schuenzel@utrgv.edu (E.S.)

**Keywords:** glioblastoma (GBM), PI3K/AKT/mTOR pathway, glycolysis, RNA-seq, Gene Set Enrichment Analysis (GSEA)

## Abstract

Glioblastoma (GBM) is the most lethal primary brain cancer that lacks effective molecular targeted therapies. The PI3K/AKT/mTOR pathway is activated in 90% of all Glioblastoma multiforme (GBM) tumors. To gain insight into the impact of the PI3K pathway on GBM metabolism, we treated U87MG GBM cells with NVP-BEZ235 (PI3K and mTOR a dual inhibitor) and identified differentially expressed genes with RNA-seq analysis. RNA-seq identified 7803 differentially regulated genes in response to NVP-BEZ235. Gene Set Enrichment Analysis (GSEA) identified two glycolysis-related gene sets that were significantly enriched (*p <* 0.05) in control samples compared to NVP-BEZ235-treated samples. We validated the inhibition of glycolytic genes by NVP-BEZ235 and examined the impact of the FOXO1 inhibitor (AS1842856) on these genes in a set of GBM cell lines. FOXO1 inhibition alone was associated with reduced *LDHA* expression, but not *ENO1* or *PKM2*. Bioinformatics analyses revealed that PI3K-impacted glycolytic genes were over-expressed and co-expressed in GBM clinical samples. The elevated expression of PI3K-impacted glycolytic genes was associated with poor prognosis in GBM based on Kaplan–Meier survival analyses. Our results suggest novel insights into hallmark metabolic reprogramming associated with the PI3K-mTOR dual inhibition.

## 1. Introduction

Cancer is the second leading cause of death in the United States. Glioblastoma multiforme (GBM) is the most fatal, neurologically destructive, and common brain tumor of the central nervous system (CNS) [1]. GBM (grade IV glioma) is highly proliferative and classified as low-grade and high-grade glioblastoma (LGG and HGG) as per WHO standards [1,2]. GBM develops from astrocytes, oligodendrocytes, or from their precursors, and represents ~80% of the total malignant tumors of the CNS [1,2]. GBM is characterized by rapid growth, aggressive invasion, high rate of recurrence, neovascularization, and poor prognosis [2,3,4]. GBM is the highest occurring malignant brain tumor in the United States and has median survival period of ~15 months [2,5,6].

Glioblastoma is difficult to treat owing to widespread invasiveness, complex surgical resection, and resistance to chemotherapy and radiation therapy [5]. Traditionally available surgical tumor resection, chemotherapy, and radiation therapies are associated with poor survival rate (14 to 18 months) [6,7]. The heterogeneous nature of GBM makes it difficult for the development of treatments against these aggressive tumors and is responsible for tumor recurrence [6]. A lack of effective treatments for GBM necessitates the development of novel molecular approaches for combating this cancer.

Cancer cells are characterized by alterations in metabolic pathways and possess resistance to cell death. Abrupt changes in glucose metabolism are perhaps the most distinguishing characteristics between malignant and normal cells. Glycolysis generates high-energy ATP from glucose to meet biosynthetic and energetic demands [8]. In GBM, cancer cells undergo the Warburg effect, a metabolic reprogramming to utilize aerobic glycolysis as the main source of energy and excrete lactate even in the presence of sufficient oxygen [9,10,11]. In GBM, glycolysis is increased compared to the normal cells [12,13]. Furthermore, oncogenes promote glycolytic metabolism in contrast to tumor suppressors [14]. Along with the Warburg effect, cell metabolism in GBM is commonly characterized by alterations in oxidative phosphorylation (OXPHOS), dysfunctional lipid metabolism, as well as other metabolic pathways [15]. 

The evolutionarily conserved phosphatidylinositol 3-kinase (PI3K)/mammalian target of rapamycin (PI3K/AKT/mTOR) pathway controls major physiological processes such as survival, proliferation, apoptosis, metabolism, motility, and growth [1,16,17]. The PI3K/AKT/mTOR pathway is often impacted in GBM by mutations in *EGFR*, *PTEN, PIK3CA, AKT* (*PKB*), and *PDGFRA* to increase pathway output [18,19]. Furthermore, increased activity of PI3K/AKT/mTOR signaling pathways in GBM is highly correlated with tumor progression, multidrug resistance, and poor prognosis [18,20]. Activation of PI3K/AKT/mTOR enhances tumor micro-vessel density and increases invasive potential of cancer cells [21]. 

PI3K/AKT/mTOR impacts glycolytic metabolism in numerous ways, including the direct activation of glycolytic enzymes. AKT phosphorylates hexokinase 2, leading to its activation. AKT also impacts numerous transcription factors, including hindering FOXO transcription factors to promote glycolysis at least in some contexts in a mechanism that involves alleviation of MYC suppression by FOXO factors [22]. AKT also promotes glucose uptake [22]. The MYC oncogene, which encodes c-MYC (or MYC) basic helix–loop–helix (and leucine zipper) transcription factor, is a critical regulator of glucose and glutamine metabolism central to the Warburg effect, and highly associated with several human cancers [23]. Novel mechanisms have been identified that may regulate glycolysis at least in part independently of PI3K. Under limiting glucose conditions, SHC3 promotes vesicle recycling of glucose transporters belonging to the GLUT/SLC2A superfamily to increase glucose uptake in GBM [24]. Given the importance of glycolysis in cancer progression, many pathways and feedback mechanisms are in place to ensure energetic demands are met [25]. Understanding variations in metabolism upon glycolytic perturbations such as upon PI3K pathway inhibition will shed light on feedback mechanisms that deter treatment efficacy.

The PI3K/AKT/mTOR pathway regulates forkhead-box type O (FOXO) transcription factors, which have a role in tumor suppression, and act in a context-dependent manner [26,27]. Three members of the FOXO family of transcription factors (FOXO-1, FOXO-3, and FOXO-4) are widely expressed in mammals and possess tumor suppressor activity by regulating apoptosis, DNA repair, oxidative stress resistance, and cell cycle arrest activity [19,27]. FOXO6 is a less conserved homolog whose expression is primarily in the brain [28]. Even though FOXO transcription factors are known to perform tumor suppression activities, recent studies identified their involvement in cancer-promoting activities. Inhibition of the PI3K/AKT/mTOR pathway can lead to increased expression of stem markers such as *OCT4* and *ALPP*, in part by activation of FOXO transcription factors [17]. In addition, FOXO transcription factors can act as stress response factors. Similarly, prolonged FOXO activity can promote resistance to chemotherapeutic treatment (doxorubicin) in breast cancer [26,29]. In neuroblastoma, expression of FOXO3 was beneficial as a prognostic marker and a biomarker to assess the efficacy of the PI3K/AKT inhibition [26]. Overexpression of FOXO3 and AKT inhibition increased apoptosis in neuroblastoma [26]. These studies suggests that the role of FOXO factors in tumor outcome is largely dependent on context. Furthermore, in the context of PI3K/AKT/mTOR inhibition in GBM, the role of FOXO transcription factors is largely unknown. 

The PI3K/AKT/mTOR pathway harbors feedback mechanisms that sustain its activity even in the presence of inhibitors. NVP-BEZ235 is a dual PI3K inhibitor that targets the catalytic subunit of PI3K and mTOR [30]. Many groups have studied this drug in contexts such as GBM and clear cell renal carcinoma [31]. For example, Heinzen et al. found that NVP-BEZ235 reduced growth and glucose consumption in GBM cell lines [32]. We previously investigated NVP-BEZ235 in the context of basal breast cancer and GBM and found that 50 nM NVP-BEZ235 enabled stem gene expression such as *OCT4* in these settings but was also associated with decreased cell growth and apoptosis induction [17,33]. To better understand the molecular underpinnings for these phenotypes, we performed RNA-seq analysis with U87MG cells treated with NVP-BEZ235. Comparative analysis of RNA-seq data followed by Gene Set Enrichment Analysis (GSEA) [34] was performed to identify significantly differentially expressed genes and biological pathways associated with PI3K/AKT/mTOR pathway inhibition in GBM. NVP-BEZ235-regulated genes were validated in numerous cell lines. Given that FOXO1 is commonly activated upon NVP-BEZ235 treatment, we examined whether FOXO1 inhibition with (AS1842856) would suppress changes in NVP-BEZ235-driven gene expression. We found that FOXO1 inhibition did suppress some of the NVP-BEZ235-directed changes in gene expression, but also had independent impacts on metabolism. We used the GEPIA2 tool to analyze the clinical importance of the identified glycolytic genes for association with patient survival, co-expression (correlation analysis), and expression analysis [35]. Our findings suggest that genes involved in glycolysis were significantly downregulated by PI3K-mTOR inhibition with NVP-BEZ235. Delineating molecular changes associated with glycolysis under PI3K-mTOR inhibition would reveal critical metabolic factors for developing novel therapeutic solutions.

## 2. Materials and Methods

### 2.1. Cell Lines, Cultures, and Drug Treatment

All cell lines used in this study were provided by the American Type Culture Collection (Manassas, VA, USA). U87MG cells were propagated in MEM (Minimal Essential Medium, Thermo Fisher, Waltham, MA). DBTRG cells were propagated in RPMI (Roswell Park Memorial Institute 1640 Medium, Thermo Fisher, Waltham, MA). LN-18, U118 MG, and LN-229 cells were propagated in DMEM (Dulbecco’s Modified Eagle Medium, Thermo Fisher, Waltham, MA, USA). Cell lines were grown in standard propagating conditions supplemented with 10% fetal bovine serum (FBS, Thermo Fisher, Waltham, MA), antibiotic (Anti-Anti, Gibco cat: 15240096, Thermo Fisher, Waltham, MA), and 5% CO_2_ at 37 °C. The dual PI3K-mTOR pathway inhibitor NVP-BEZ235 and FOXO1 inhibitor AS1842856 were acquired from Sigma-Aldrich (Saint Louis, MO, USA) and Calbiochem (Danvers, MA, USA), respectively. NVP-BEZ235 and AS1842856 were utilized in final concentrations at 50 nM (or 1 μM) and 200 nM, respectively. Control samples were treated with DMSO.

### 2.2. RNA Extraction and RNA Sequencing

U87MG cells were plated at a density of 2700 cells per mL in complete media as described above. Then, 50 nM NVP-BEZ235 or DMSO vehicle were added to U87MG cells for four days. Next, total RNA was extracted from the treated and control samples using the Qiagen MiniPrep RNAeasy kit (Hilden, Germany). Three biological replicates of control and NVP-BEZ235-treated cell lines were used for the RNA extraction. The quality of the extracted RNA was analyzed using NanoDrop spectrophotometer (Thermo Fisher, Waltham, MA, USA). RNA-seq was performed by Novogene Corporation Inc. (Sacramento, CA, USA). 

### 2.3. Bioinformatics Analysis

RNA-seq analyses including mapping to human genome, sequence assembly, and identification of differentially expressed genes (DEGs) were performed by the Novogene Corporation Inc. (Sacramento, CA, USA). The original image data file from high-throughput sequencing (Illumina, San Diego, CA, USA) was transformed into sequenced reads (called raw data or raw reads) by CASAVA base recognition (base calling). Raw data were stored in FASTQ (fq) format files, which contain sequences of reads and corresponding base quality. STAR software was used to accomplish the mapping. The abundance of transcript reflects gene expression level directly. In this RNA-seq experiment, gene expression level was estimated by the abundance of transcripts (count of sequencing) that mapped to genome or exon. Read counts were proportional to gene expression level, gene length, and sequencing depth. FPKM (short for the expected number of fragments per kilobase of transcript sequence per million base pairs sequenced) was the most common method of estimating gene expression levels, which takes the effects into consideration of both sequencing depth and gene length on counting of fragments. For the samples with biological replicates, differential expression analysis of two conditions/groups was performed using the DESeq2 R package.

RNA-seq data were deposited to the GEO database (accession: GSE179667). The RNA-seq data were analyzed to identify differentially expressed genes (DEGs) associated with NVP-BEZ235 treatment. DEGs between NVP-BEZ235-treated and control cell lines were investigated using the Gene Set Enrichment Analysis (GSEA) to identify enriched biological pathways defined by the set of genes between the two treatments. Gene sets were downloaded from the Molecular Signatures Database (MSigDB) (https://www.gsea-msigdb.org/gsea/msigdb/index.jsp, accessed on 27 September 2020) for GSEA analysis. GSEA was performed by setting the ENSEMBL Gene ID v7.0 Chip platform, 1000 number of permutations, and weighted enrichment statistics [34]. Heatmaps for gene expression counts in control and NVP-BEZ235 treatments were generated using the MORPHEUS online tool (https://software.broadinstitute.org/morpheus/, accessed on 5 February 2021). Kaplan–Meier survival analysis, gene expression box plots, and Pearson correlations were calculated from the GEPIA2 online tool [35]. The GO enrichment analysis was performed using the gene ontology resource (http://geneontology.org/, accessed on 10 February 2021).

### 2.4. Quantitative Real-Time PCR (qRT-PCR)

Log-phase cancer cell lines were treated with 1 μM NVP-BEZ235 for five days. This dose more fully inhibits PI3K [36]. Extracted total RNA was used to prepare cDNA using Superscript Reverse Transcriptase II (Invitrogen, Carlsbad, CA). Primers spanning the coding sequences for the genes were designed using Primer3 (v0.4.0) (https://bioinfo.ut.ee/primer3-0.4.0/, accessed on 30 September 2020) and ordered from Sigma-Aldrich (Saint Louis, MO). Amplification and expression of genes were performed using the Applied Biosystems StepOne Real-Time PCR System (Foster City, CA, USA). Four technical replicates were used for the gene amplification and quantification. *ACTIN* (*ACTB* gene) was used as housekeeping gene for relative normalization and quantification performed by the 2^−ΔΔCT^ method provided in the Applied Biosystems PCR machine [37]. For serum-free medium, log-phase cells were starved for five days. Briefly, the cells were washed twice with serum-free media and kept in serum-free media with selective antibiotics (MEM for U87MG and DMEM for LN18). The drug concentration was 1 μM for a five-day treatment. PCR data were exported, and analysis was performed using MS Excel and R project for statistical computing for average, standard error, and Student’s *t*-test calculation or Tukey’s test.

### 2.5. Western Blot

Log-phase cancer cell lines were treated with 1 μM NVP-BEZ235 for five days. Total protein was obtained from indicated cells by rinsing cells with 1XPBS (phosphate buffered saline) followed by directed lysis in 2× sample buffer (125 mM Tris-HCL at pH 6.8, 2% sodium dodecyl sulfate (SDS), 10% 2-mercaptoethanol, 20% glycerol, 0.05% bromophenol blue, 8 M urea); 2× sample buffer was added to each well and cells were scraped with a cell scraper. The lysate was collected from each well, placed into a 1.5 mL microcentrifuge tube, and heated for 10 min at 95 °C in a dry-bath heat block. Protein lysates were separated by sodium dodecyl sulfate–polyacrylamide gel electrophoresis (SDS-PAGE) at 100 V for 1 h. Resolved proteins were then transferred onto a polyvinylidene fluoride (PVDF) membrane for an hour and 30 min, then blocked in a 5% milk solution (Carnation powdered milk) and 1× Tris-buffered saline with Tween 20 (TBST) for an hour. Membranes were incubated with indicated primary antibody overnight at 4 °C then washed for 20 min with TBST in 5-min intervals. The blot was then incubated with secondary antibody for 1.5 h. Membranes were washed for 20 min in 5-min intervals and allowed to develop using SuperSignal West Dura Extended Duration Substrate luminol solution (Pierce Biotechnology, Waltham, MA, USA) for 5 min. A Bio Rad ChemDoc XRS+ Molecular Imager was utilized for protein detection (Bio Rad, Hercules, CA, USA). Data were analyzed with NIH Image J. Antibodies were obtained from Cell Signaling Technologies (Danvers, MA, USA): LDHA (C4B5), PKM2 (D78A4) XP, Enolase-1 Antibody #3810, phospho-AKT serine 473 (9271S) and total AKT (4691S). GAPDH (G-9) was obtained from Santa Cruz Biotechnology Inc., Dallas, TX, USA. Beta-Actin antibody (clone AC-74, cat: A2228) was obtained from Sigma-Aldrich (Saint Louis, MO, USA) and utilized at a 1:2000 dilution in TBST with 5% non-fat dried milk.

### 2.6. Quantitative Glutamate Assay

The quantification of glutamate in control (DMSO) and NVP-BEZ235-treated GBM cells lines was performed using the Glutamate Colorimetric Assay kit (#K629-100, BioVision Inc., Milpitas, CA, USA) as per the manufacturer’s protocol. Log-phase U87MG cells were treated with 1 μM NVP-BEZ235 for five days. The 1 mM glutamate standard was prepared and added to a 96-well plate to generate two replicates of 0, 2, 4, 6, 8, and 10 nmol/well standard concentrations. Absorbance for glutamate standards and samples were measured at 450 nm using iMark Microplate Absorbance Reader (Bio-Rad, Hercules, CA). Standard and sample absorbances were corrected relative to the background control to visualize the standard curve using regression equation. Glutamate concentrations were estimated from the regression equation and using the formulae C = Sa/Sv provided by the manufacturer’s protocol.

### 2.7. NAD+/NADH Cell-Based Assay

The nicotinamide adenine dinucleotide (NAD+) in normal and GBM cells were measured using Cayman’s NAD+/NADH cell-based assay kit as per the manufacturer’s protocol. Briefly, the NAD+ standards were prepared in the range of 0 to 1000 nM as per the manufacturer’s protocol. Log-phase U87MG cells were treated with 1 μM NVP-BEZ235 for five days. Cells were washed, centrifuged, and incubated for 90 min after adding 100 µL reaction solution to each well. The absorbance for NAD+ standards and samples were measured at 450 nm using iMark Microplate Absorbance Reader (Bio-Rad, Hercules, CA, USA). Standard and sample absorbances were corrected relative to the background control to visualize the standard curve with regression equation. Total cellular NAD+ concentrations were calculated using the standard curve equation as per the manufacturer’s protocol. 

### 2.8. Glucose Uptake and Lactate Secretion Assays

Glucose Uptake-Glo^TM^ and Lactate-Glo ^TM^ (Promega, Madison, WI, USA) assay kits were used to perform the glucose uptake and lactate secretion assays, respectively, as per the manufacturer’s instructions. Both assays were performed with or without FBS with similar results. For glucose uptake assays, log-phase U87MG cells were treated with 1 μM NVP-BEZ235 for 48 h under starvation conditions (without FBS). After treatment, 36,000 cells were washed with PBS and incubated with 2-deoxy-D-glucose (2DG) for 10 min at room temperature. Luminescence was recorded using a 20/20n luminometer from Turner Biosystems (Sunnyvale, CA, USA). Samples were examined in quadruplicate in replicate experiments.

For lactate secretion assays, log-phase U87MG cells were treated with 1 μM NVP-BEZ235 for 48 h under starvation conditions (without FBS). Then, 3 μL of culture media were placed into 97 μL of PBS and were incubated with the Lactate Detection Reagent for 60 min at room temperature. The luminescence was recorded using the 20/20n luminometer from Turner Biosystems. Cell numbers were counted for each sample and were used to normalize luminescence readings for comparisons. Samples were examined in quadruplicate in replicate experiments.

## 3. Results

### 3.1. NVP-BEZ235 Treatment Reduced Glycolytic Gene Expression in GBM Cells

**Figure 1:**
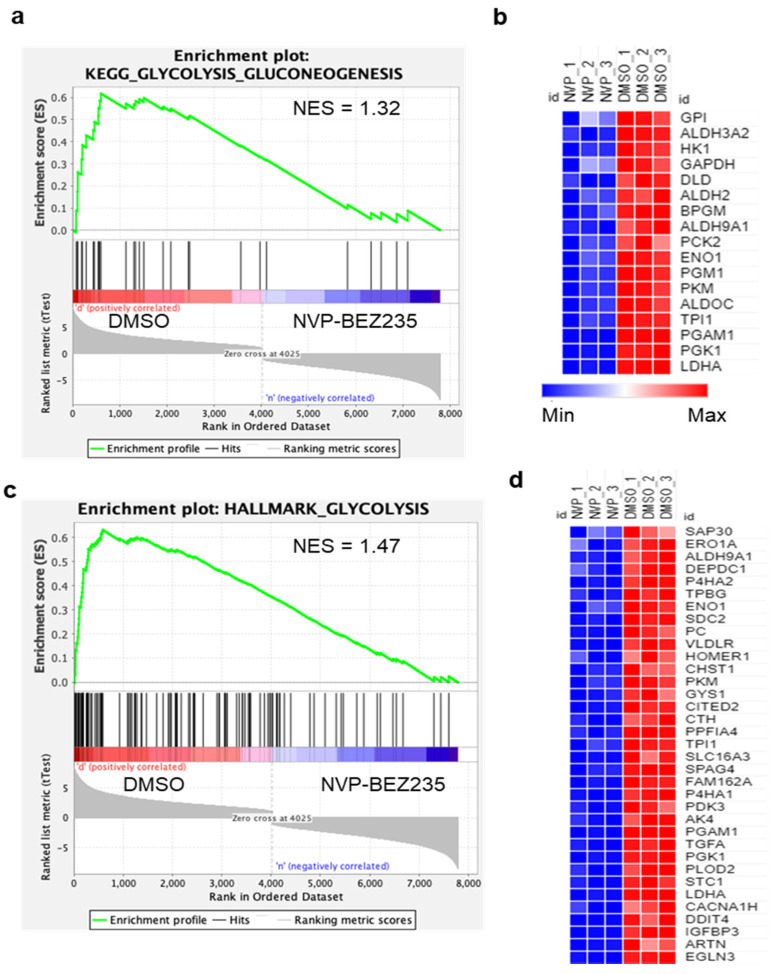
Glycolytic genes are reduced by NVP-BEZ235 treatment in U87MG cells. GSEA generated enrichment plots and generated heatmaps depicting the enrichment of sample phenotypes, control (DMSO) and NVP-BEZ235-treated (50 nM for four days in U87MG cells), in priori defined glycolysis gene sets. (**a**) KEGG glycolysis gluconeogenesis gene set, (**b**) heatmap of 17 core enriched genes KEGG_GLYCOLYSIS_GLUCONEOGENESIS gene set (**c**), Hallmark glycolysis gene set, (**d**) heatmap of 35 core-enriched genes in HALLMARK_GLYCOLYSIS gene set. The profile of enrichment score (ES) and rank ordered list for enriched genes are shown. Both gene sets were significantly enriched in control samples compared to NVP-BEZ235 treatment samples *p <* 0.05.

RNA sequencing was performed from U87MG cells treated for four days with 50 nM NVP-BEZ235 (dual PI3K-mTOR pathway inhibitor) or vehicle control with three biological replicates of each condition. This drug dose was employed in our previous studies (and others) and is not a complete inhibition of PI3K-mTOR in U87MG cells [31,33]. The four-day timepoint allowed us to obtain high-quality RNA for sequencing. Initial bioinformatics analyses included quality filtering. Genome mapping followed by comparative gene expression analysis between NVP-BEZ235-treated and control samples led to the identification of 7803 differentially expressed genes (DEGs). The 7803 DEGs (native features) obtained from the RNA-seq analysis were used for GSEA [34,38]. Analysis with the Molecular Signatures Database curated Hallmark gene sets revealed that seven gene sets were significantly enriched in control samples (compared to NVP-BEZ235-treated samples), including HALLMARK_HYPOXIA, HALLMARK_GLYCOLYSIS, and HALLMARK_HEME_METABOLISM (Table S1). Several gene sets were significantly enriched in NVP-BEZ235-treated samples: HALLMARK_COMPLEMENT, HALLMARK_PANCREAS_BETA_CELLS, and HALLMARK_COAGULATION (Table S2). We further investigated the impact of NVP-BEZ235 on glycolytic gene expression given the robust enrichment of these genes in control samples (ES = 63, *p <* 0.001, Table S1). GSEA was carried out for the glycolysis C1 (hallmark gene set) and C2 gene sets (curated gene sets), including the KEGG_GLYCOLYSIS_GLUCONEOGENESIS and HALLMARK_GLYCOLYSIS collections available in the MSigDB (https://www.gsea-msigdb.org/gsea/msigdb/genesets.jsp, accessed on 27 September 2020) [39]. GSEA indicated that glycolytic gene sets were significantly enriched in the control (DMSO samples) compared to NVP-BEZ235-treated samples (*p <* 0.05) in KEGG_GLYCOLYSIS_GLUCONEOGENESIS and HALLMARK_GLYCOLYSIS (Figure 1a,c and Table S3). 

KEGG_GLYCOLYSIS_GLUCONEOGENESIS and HALLMARK_GLYCOLYSIS gene sets were further analyzed for GSEA core enrichment; we found that 17 and 35 genes, respectively, were significantly downregulated in GBM cell lines in response to NVP-BEZ235 treatment (Table S3). To represent the expression pattern of a large number of core enriched genes, we created the heatmap for enriched genes in KEGG_GLYCOLYSIS_GLUCONEOGENESIS and HALLMARK_GLYCOLYSIS based on the expression values of the genes in control and treated samples (Figure 1b,d). Overall, a large number of glycolysis-related genes were downregulated in response to NVP-BEZ235 treatment in GBM cell lines (Figure 1b,d).

**Figure 2:**
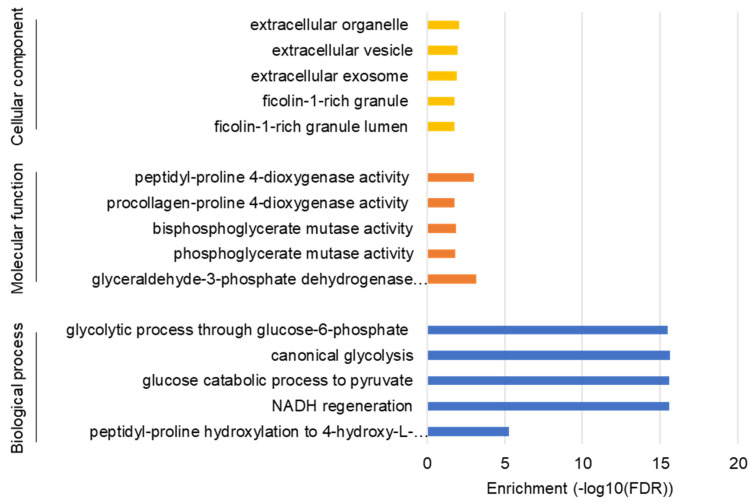
GO enrichment analysis for biological process, molecular function, and cellular components. The false discovery rate (FDR) values on a minus log10 scale were used for enrichment measurement.

To understand the comprehensive biological functions of NVP-BEZ235-regulated genes, we performed GO enrichment analysis for biological processes, molecular functions, and cellular components. Glycolysis-related genes were highly enriched for glucose catabolism, canonical glycolysis, and other glycolytic processes (Figure 2). The GO terms for glyceraldehyde-3-phosphate dehydrogenase activity and peptidyl-proline 4-dioxygenase activity were also highly enriched under molecular functions (Figure 2). Similarly, ficolin-1-rich granule and extracellular organelle were enriched under cellular component categories (Figure 2). The detailed GO terms are provided in Table S4.

### 3.2. NVP-BEZ235 Treatment Reduced Glycolytic Genes in Numerous GBM Cell Lines

**Figure 3:**
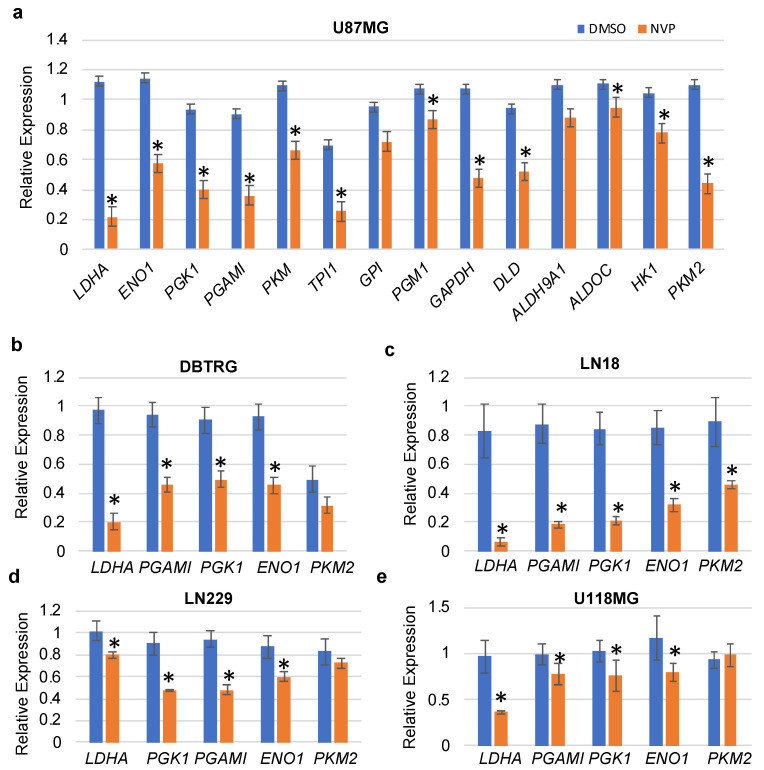
qRT-PCR validation of the glycolytic genes in various cell lines. (**a**) U87MG, (**b**) DBTRG, (**c**) LN18, (**d**) LN229, and (**e**) U118MG. The relative expression of the genes (compared to *ACTB*, encoding actin) was analyzed in control and NVP-BEZ235 (NVP) samples (1 μM treatment for 5 days). The error bars represent the standard deviation of the genes (n = 4). Asterisks (*) represent the significantly expressed genes (*p <* 0.05) as compared to control (DMSO). Significance was determined by Student’s *t*-test.

To validate differentially expressed glycolysis-related genes obtained from RNA-seq data, we performed qRT-PCR. Primers for selected genes were designed using the Primer3 (v0.4.0) online tool (Table S5). qRT-PCR analysis showed that selected glycolysis-related genes were significantly downregulated in control (DMSO) as compared to NVP-BEZ235 (Figure 3a). Validation of differential gene expression by qRT-PCR also built confidence and reliability in the RNA-seq analysis.

To determine whether NVP-BEZ235 impacted glycolytic genes in other cell lines in addition to U87MG cells, we investigated the expression of these genes in a panel of GBM cell lines including DBTRG, LN18, LN229, and U118MG (1 μM NVP-BEZ235 for five days, Figure 3b–e). Results indicated that the *LDHA* gene was consistently downregulated in all cell lines in response to NVP-BEZ235 treatment (Figure 3b–e). Similarly, *ENO1* was downregulated in all cell lines in response to NVP-BEZ235 treatment (Figure 3b–e). *GAPDH* was slightly upregulated in the DBTRG GBM cell line (data not shown). 

**Figure 4:**
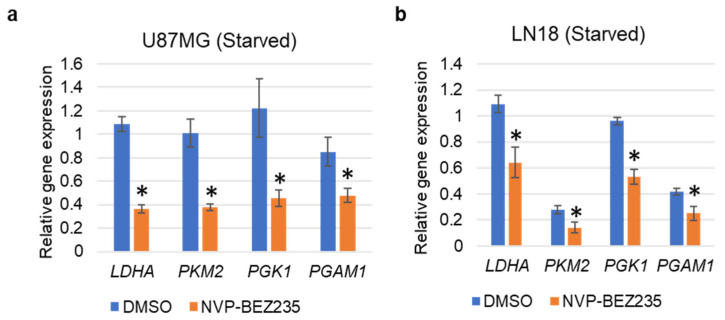
qRT-PCR validation of the glycolysis-related genes under serum starvation in (**a**) U87MG, and (**b**) LN18 cell lines grown in serum-free medium. The relative expression of the genes (compared to *ACTB*, encoding actin) was analyzed in control DMSO and NVP-BEZ235 samples (five days 1 μM NVP-BEZ235 with no FBS). The error bars represent the standard deviation of the genes (n = 4). Asterisks (*) represent the significantly expressed genes (*p <* 0.05) as compared to control (DMSO). Significance was determined by Student’s *t*-Test.

In addition to utilizing FBS-supplemented medium, we also performed the validation of the selected genes with serum starvation conditions in U87MG and LN18 GBM cell lines. Results indicated that *LDHA*, *PKM2*, *PGK1*, and *PGAM1* were significantly downregulated in both U87MG and LN18 GBM cell lines in response to NVP-BEZ235 as compared to the control DMSO (Figure 4a,b).

**Figure 5:**
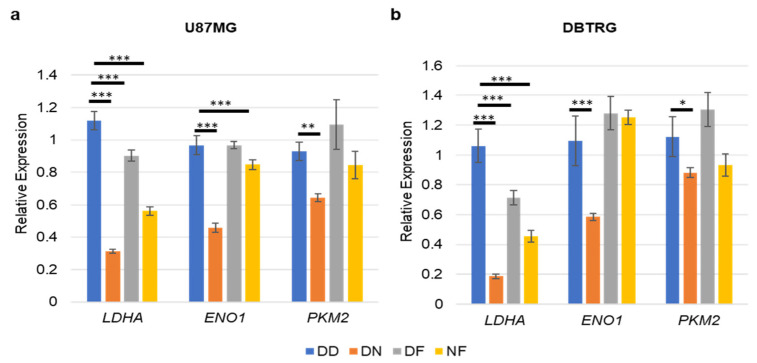
FOXO1 inhibition partially suppressed NVP-BEZ235 induction of glycolytic genes. (**a**) U87MG and (**b**) DBTRG. The relative expression of the genes was analyzed in DD-, DN-, DF-, and NF-treated samples. DD: DMSO; DN: DMSO+NVP-BEZ235 (1 μM); DF: DMSO+FOXOi (1 μM AS1842856); NF: NVP-BEZ235+FOXOi (1 μM of each drug). The error bars represent the standard deviation of the genes. The *p*-values were calculated by one-way ANOVA followed by Tukey’s test (*, *p <* 0.05; **, *p <* 0.01, ***, *p <* 0.001).

The PI3K pathway was previously found to promote glycolytic gene expression in U87MG cells that harbored exogenous constitutively active *EGFR-vIII* [40]. Specifically, the reduction of *RICTOR*, a defining component of mTORC2, decreased glycolytic gene expression in a FOXO1-dependent manner. FOXO1 induced the expression of *miR-34c* to decrease *MYC* expression in U87MG cells that expressed activated *EGFR-vIII*. To determine whether a similar mechanism was employed in cells treated with dual PI3K pathway inhibitor NVP-BEZ235, we assessed the combined impact of PI3K-mTOR inhibition (NVP-BEZ235 1 μM) and FOXO1 inhibition (FOXO1 inhibitor AS1842856 1 μM) on glycolytic gene expression in U87MG and DBTRG GBM cancer cell lines. In both cell lines, *LDHA* was reduced with NVP-BEZ235 or FOXO1 inhibition with AS1842856 (Figure 5a,b), suggesting that both PI3K and FOXO1 promoted expression of this gene. Of note, combined NVP-BEZ235 and AS1842856 led to increased *LDHA* and *ENO1* compared to NVP-BEZ235 alone in U87MG cells, suggesting that the loss of FOXO1 suppressed the reduction of these genes in NVP-BEZ235-treated cells (Figure 5a,b).

**Figure 6:**
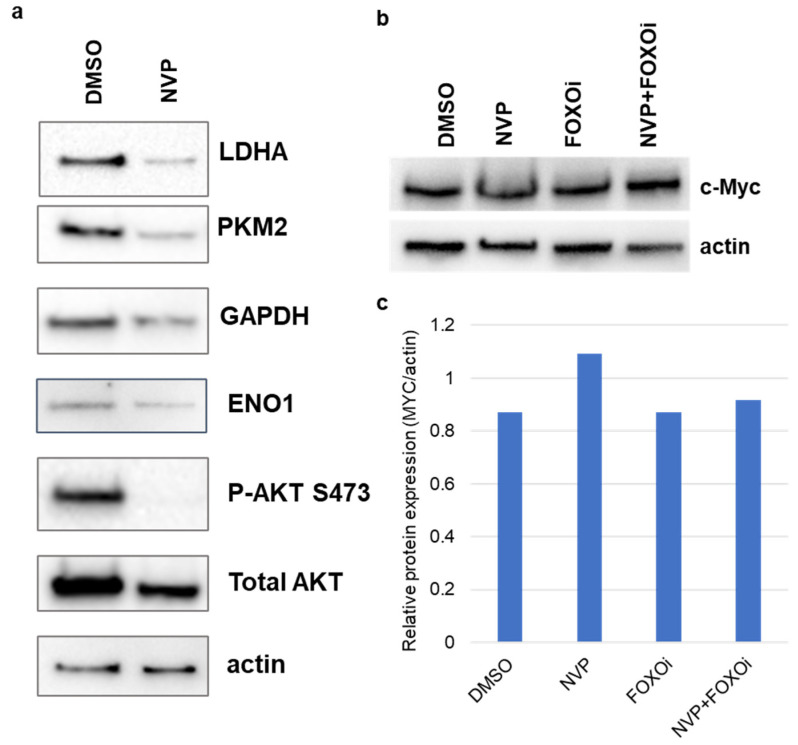
Expression changes of glycolysis-related proteins. Protein expression was verified using Western blot analysis in U87MG in (**a**) DMSO and 1 μM NVP-BEZ235 (NVP) samples, (**b**) c-Myc protein expression was verified using Western blot analysis in DMSO, NVP, FOXOi (AS1842856), and NVP+FOXOi samples (1 μM of each drug). Actin was used as the loading control. (**c**) Western blot for c-Myc was quantified using the Image Lab Software (Bio-Rad).

We verified the protein expression of the glycolytic genes in U87MG cells treated with 1 μM NVP-BEZ235 (NVP) for five days. Expression levels of LDHA, PKM2, and GAPDH were higher in DMSO-treated samples as compared to NVP-BEZ235-treated samples (Figure 6a). Furthermore, we assessed the C-Myc protein expression under the FOXOi (AS1842856 1 μM) and combined inhibition (NVP+FOXOi) and found that c-Myc expression was not impacted by FOXOi or NVP+FOXOi as compared to vehicle control DMSO (Figure 6b,c). The expression of c-Myc was slightly higher in NVP-BEZ235-treated samples as compared to control DMSO (Figure 6b,c).

### 3.3. NVP-BEZ235 Treatment Led to Reduced NAD+ and Glutamate in U87MG Cells

**Figure 7:**
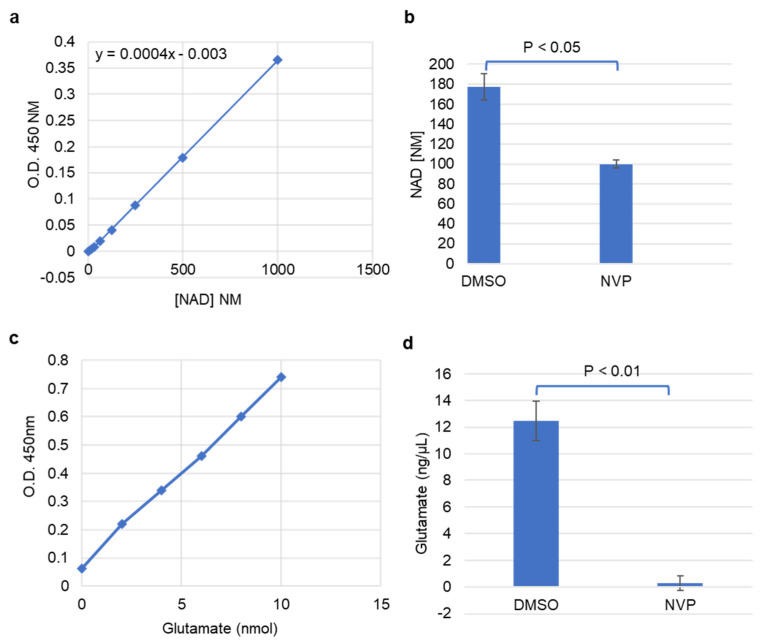
NAD+ and glutamate were reduced with NVP-BEZ235 treatment. (**a**) Absorbance of NAD+ concentrations at 450 nm. (**b**) The estimated concentration of NAD+ in control DMSO and 1 μM NVP-BEZ235 samples. (**c**) Absorbance of glutamate concentrations at 450 nm. (**d**) The estimated concentration of glutamate in DMSO and 1 μM NVP-BEZ235-treated samples. The error bars represent the standard deviation of the samples. Significance was determined by Student’s *t*-test.

Given that glycolytic genes were reduced upon NVP-BEZ235 treatment of U87MG cells, we examined whether a concomitant change in metabolism could be observed. Functional assays were performed to assess the impacts of NVP-BEZ235 on metabolism, including cellular NAD+ levels, glutamate levels, glucose uptake, and lactate secretion. In the Warburg effect, high levels of NAD^+^/NADH are produced as a result of increased glycolytic activity in cancer cells [41]. We found that the NAD+ concentration was significantly higher in DMSO samples as compared to the 1 μM NVP-BEZ235-treated cells, suggesting that inhibition of PI3K-mTOR reduced glycolytic metabolism (Figure 7a,b). 

Glutamate is another important metabolite associated with cancer progression that can promote glycolysis in certain contexts. We investigated changes in glutamate concentration in control and NVP-BEZ235-treated cell lines. The colorimetric glutamate assay kit was used to analyze the concentration of glutamate in the samples. Glutamate concentration was significantly higher in control samples as compared to the 1 μM NVP-BEZ235-treated cells, suggesting that inhibition of PI3K-mTOR causes a reduction in glutamate (Figure 7c,d). This observation highlights that glutamate is diminished tenfold by PI3K-mTOR inhibition.

### 3.4. Glucose Uptake and Lactate Secretion Were Reduced under NVP-BEZ235 Treatment in U87MG Cells 

**Figure 8:**
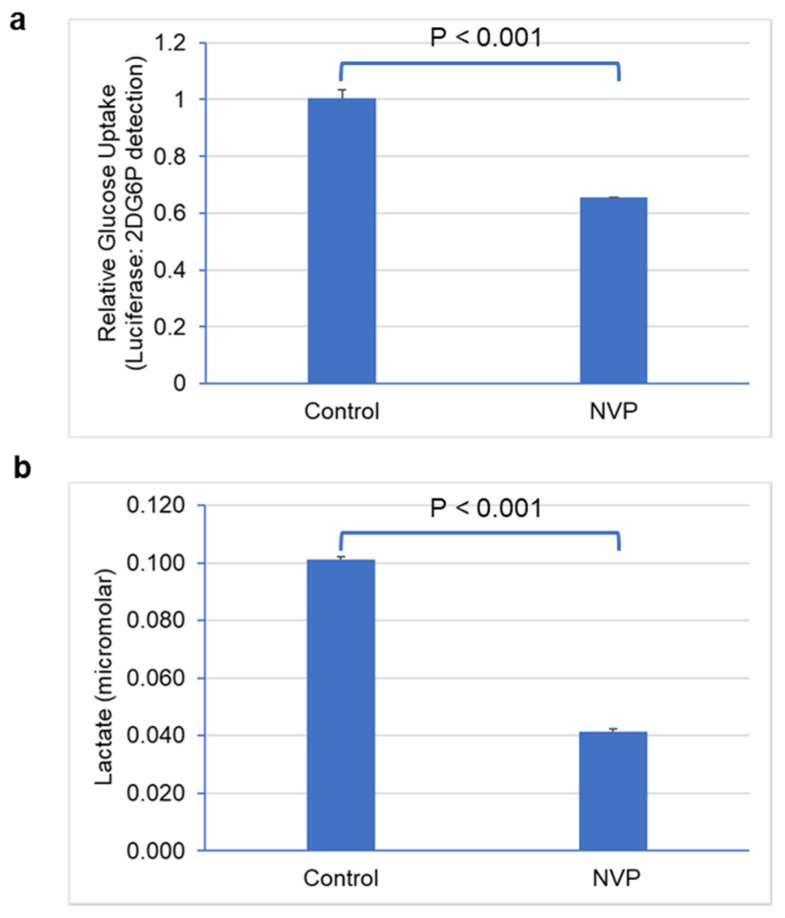
NVP-BEZ235 treatment reduced glucose uptake and lactate secretion. (**a**) Glucose uptake and (**b**) lactate secretion in control DMSO and NVP-BEZ235-treated (1 μM) samples were measured with luminescence assay. The error bars represent the standard deviation of the samples. Significance was determined by Student’s *t*-test.

To further understand the regulation of glycolytic metabolism in NVP-BEZ235-treated cell lines, we measured glucose uptake and lactate secretion. We observed that glucose uptake was significantly lower (*p <* 0.001) in NVP-BEZ235-treated samples in comparison to control DMSO (Figure 8a), consistent with work by Heinzen et al. [32]. Similarly, lactate secretion was significantly reduced (*p <* 0.001) in NVP-BEZ235-treated samples (Figure 8b). These results indicate that glycolytic metabolic reprogramming was hampered by NVP-BEZ235 in GBM cell lines.

### 3.5. Glycolytic Genes Are Associated with Poor Prognosis in GBM 

**Figure 9:**
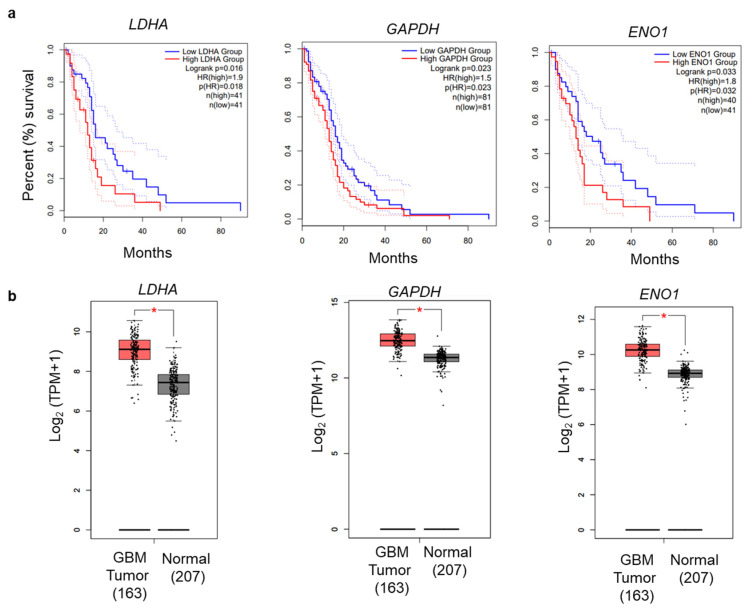
Overall survival analysis of glycolysis-related genes. (**a**) OS Kaplan–Meier analysis was performed using the GEPIA2 online application. The solid and dotted lines represent the survival curve and 95% confidence interval, respectively. (**b**) Differential expression boxplot of genes (*LDHA, GAPDH,* and *ENO1*) in GBM in The Cancer Genome Atlas (TCGA) database obtained from GEPIA2 [35].

To ascertain the potential impacts of glycolytic genes on GBM prognosis, we investigated these genes for association with patient survival in clinical samples. Data from GEPIA2 were analyzed to construct box plots and perform Kaplan–Meier analyses [35]. Among the enriched genes, elevated expression of *Lactose Dehydrogenase (LDHA), Glyceraldehyde-3-Phosphate Dehydrogenase* (*GAPDH*), and *Enolase 1* (*ENO1)* were significantly associated (log-rank *p <* 0.05) with worse survival (Figure 9a). Furthermore, we examined the expression of these genes from the GEPIA2 online tool, which has an expression database based on The Cancer Genome Atlas (TCGA) database. The comparison of expressions of *LDHA*, *GAPDH,* and *ENO1* showed overall higher expression in tumor samples as compared to the normal samples (Figure 9b). Pearson correlation analyses of the glycolysis-related genes also showed significant correlation for glycolytic genes in GBM (Table 1). Therefore, our systematic gene expression analysis obtained from RNA-seq provides a confidence in selecting genes for further genetic analysis and biological interpretation.

## 4. Discussion

GBM is the most malignant, aggressive, and lethal brain cancer worldwide, with a survival rate of less than 18 months both in treated and untreated patients. Altered glycolytic metabolism is a characteristic of GBM, where glucose is converted into lactate in the presence of oxygen for tumor cell growth, while generating ATP to meet energetic demands [25,42]. In addition, glycolysis takes place at a higher rate in GBM than in normal cells [43]. The oncogenic PI3K/AKT/mTOR pathway regulates metabolic networks and promotes aerobic glycolysis in favor of tumor cells [22]. The genetic basis of aerobic glycolysis and its regulation of the PI3K/AKT/mTOR pathway are not completely understood. Hence, decoding the key gene regulatory elements involved in glycolytic metabolism is of utmost importance for developing therapeutic treatments for GBM.

Advanced genome and transcriptomic sequencing technologies accelerated cancer research for developing precision medicine and targeted therapies. However, how cancer cells in GBM alter glycolytic metabolism and how inhibition of PI3K/AKT/mTOR alters glycolytic gene expression remained unclear. In this study, we investigated the regulation of glycolysis-related genes by comparing the transcriptomic profiles in U87MG cells using RNA-seq under NVP-BEZ235 and DMSO treatments. RNA-seq analysis identified 7803 DEGs regulated by the NVP-BEZ235 treatment. GSEA on multiple glycolysis-related gene sets identified the enrichment of 52 genes (*p <* 0.05) which were highly downregulated in response to NVP-BEZ235 (Figure 1). The analysis identified several enriched functional categories involved in glycolytic metabolism (Figure 2).

In addition to NVP-BEZ235, we also investigated the effect of FOXO1 inhibition with AS1842856 on glycolytic gene expression. We reported the decrease in *LDHA* gene expression in two GBM cell lines (U87MG and DBTRG) with NVP-BEZ235 and/or AS1842856 (Figure 5). FOXO1 inhibition did, however, at least in part suppress NVP-BEZ235-induced gene expression (Figure 5). In additional to canonical roles in PI3K (involving negative regulation by the PI3K pathway), FOXO1 had a positive impact on *LDHA* expression. Therefore, the role of FOXO1 in metabolism in U87MG cells is complex. In line with the positive impact of FOXO1 on *LDHA*, FOXO factors can be associated with cancer-promoting activities and even cause resistance to Temozolomide (TMZ) [17,44,45]. Previous reports have shown that a FOXO1 homolog (FOXO3) contributed TMZ resistance in GBM through promoting beta-catenin nuclear localization [45]. Given that FOXO1 and FOXO3 are at least in part functionally redundant, it is conceivable that FOXO1 may also promote WNT signaling, leading to TMZ resistance [46]. Understanding the molecular mechanisms underlying TMZ resistance in GBM is essential to develop new targeted therapies. 

C-Myc is a potent oncogene and is overexpressed in many human cancers [47]. Previous studies reported that FOXO1 impacted genes involved in glycolysis via down-regulation of c-MYC [27,48]. Interestingly, we found that NVP-BEZ235 alone led to increased expression of c-Myc protein as compared to control DMSO, FOXO1 inhibition (FOXOi with AS1842856 treatment), and combined inhibition (NVP-BEZ235 and AS1842856), which did not impact c-MYC in wild-type U87MG cells (Figure 6b,c). Thus, additional mechanisms aside from c-MYC regulation are utilized on the PI3K/AKT/mTOR pathway to promote glycolysis.

Kaplan–Meier survival analyses showed that high expression of glycolysis-related genes (*LDHA, GAPDH*, and *ENO1*) had a significant correlation with poor survival in GBM (Figure 9). LDHA is a vital enzyme involved in the anaerobic metabolic pathway, which catalyzes the conversion of pyruvate to lactate and NADH to NAD+ collaterally [43,49]. LDHA overexpression promotes cancer progression by providing lactate as a nutrient and acidifying the tumor microenvironment. LDHA is associated with increased levels of glycolysis in cancer cells [50]. *PKM2* encodes the rate-limiting enzyme in glycolysis that catalyzes the conversion of phosphoenolpyruvate to pyruvate. PKM2 regulates cancer cell metabolism and contributes to the growth of glioma cells [51]. Similarly, two other genes, *GAPDH* and *ENO1,* promote cancer progression in GBM [43]. The qRT-PCR analysis revealed that *LDHA, GAPDH*, and *ENO1* were decreased by NVP-BEZ235 treatment in U87MG cells (Figure 3a). Similarly, these genes followed similar expression patterns across different GBM cell lines (Figure 3b–e). We also validated the expression of these genes under serum-free medium in U87MG and LN18 GBM cell lines (Figure 4). This highlights the crucial role of glycolysis-related genes in GBM malignancy. In line with qRT-PCR analysis, glycolytic protein expression was higher in DMSO control samples compared to NVP-BEZ235-treated samples (Figure 6).

In addition to the energy requirements, cancer cells prefer glycolysis to generate intermediate biosynthetic compounds, such as amino acids, nucleic acids, lipids, and lactate, and for maintaining the NAD+/NADH redox balance [50]. The growth of the cancer cells in GBM is dependent on the availability of glutamine, which is involved in the biosynthesis of nucleotides and amino acids [25]. Glutamate is the precursor for glutamine. In GBM, glutamine synthetase (GS) catalyzes the conversion of glutamate to glutamine to meet the increased demand for glutamine [25,52]. Furthermore, glutamate secretion is enhanced under GBM conditions concomitant with increased expression of GS [52]. We observed a significant reduction in glutamate (tenfold) in NVP-BEZ235-treated samples compared to the DMSO-treated controls (Figure 7c,d). Along with glutamine, NAD+ metabolite is critical for glycolytic metabolism and tumorigenesis. Reduction in NAD+ levels led to the impairment of cancer cell growth and proliferation [53,54]. We report that NAD+ levels were significantly suppressed in NVP-BEZ235-treated cell lines (Figure 7a,b). Furthermore, in GBM, the glycolytic rate is highly increased [42], which causes increased uptake of glucose and lactate secretion. Work by Ansari et al. demonstrated that low glucose conditions (0.3g/L) reduced the expression of *MIR-451* in GBM cells due to diminished OCT1 (Octamer binding transcription factor 1) activity [55]. Ansari et al. highlight the importance of glucose levels in determining glycolytic rate. Our RNA-seq data did not have a significant change in *MIR-451* expression with NVP-BEZ235 treatment (data not shown). However, our experiments were performed with constant levels of glucose in the media (0.9g/L glucose). Our work builds upon that of Heinzen et al., who found that NVP-BEZ235 treatment led to reduced oxygen consumption and glucose consumption in GBM cells [32]. Our work revealed that NVP-BEZ235 treatment not only reduced glycolytic gene expression but also led to dramatic functional consequences in metabolic reprogramming, as evidenced by reduced cellular NAD+, glutamate, glucose uptake, and lactate secretion in U87MG cells. 

In summary, PI3K-mTOR inhibition with NVP-BEZ235 led to decreased levels of glycolytic genes, which have potential roles in GBM pathogenesis and may inform the development of novel targeted therapies for this cancer. The precise mechanisms employed by NVP-BEZ235 to impact glycolytic genes appear to be diverse and independent of MYC in the wild-type U87MG context.

## 5. Conclusions

Through comparative transcriptomic analysis, we found that glycolysis-related genes were significantly downregulated by PI3K-mTOR inhibition by NVP-BEZ235 in GBM cells. GSEA coupled with survival analyses identified key genes including *LDHA*, *GAPDH,* and *ENO1* involved in glycolytic metabolism. The molecular validation of these genes confirmed our bioinformatics results. We also reported the differential responses of glycolytic genes and c-Myc under combinatorial drug treatments that included FOXO inhibition, which showed that FOXO1 activation alone could not explain observed glycolytic changes upon NVP-BEZ235 treatment. Furthermore, we found a significant reduction in NAD+ and glutamate metabolites as well as decreased glucose uptake and lactate secretion under PI3K-mTOR inhibition (NVP-BEZ235 treatment), suggesting decreased glycolytic metabolism in GBM. These findings and the knowledge generated from this study will contribute towards advancing precision medicine for GBM. Glycolysis may serve as a barometer of NVP-BEZ235 treatment efficacy.

## Figures and Tables

**Table 1:** Pearson correlation analysis of glycolysis-related genes in GBM.

Gene	Gene	Correlation Coefficient (R)	*p*-Value
*LDHA*	*ENO1*	0.56	<0.05
*LDHA*	*GAPDH*	0.64	<0.05
*ENO1*	*GAPDH*	0.55	<0.05

## Data Availability

All cell lines and additional data prepared from this work are available upon request. RNA-seq data were deposited into the NCBI Geo database (accession: GSE179667).

## Supplementary Materials

The following are available online at https://www.mdpi.com/article/10.3390/cells10113065/s1, Table S1 GSEA results enrichment in control (DMSO samples) compared to NVP-BEZ235 treatment samples, Table S2: GSEA results for enrichment in NVP-BEZ235 treatment samples compared to control samples, Table S3: GSEA analysis output for KEGG_GLYCOLYSIS_ GLUCONEOGENESIS and HALLMARK_GLYCOLYSIS gene sets, Table S4: Detailed GO analysis table for enriched GO terms for biological process, molecular function, and cellular components, Table S5: List of forward and reverse primers of glycolysis-related genes and actin.
